# Changes in Georgia Restaurant and Bar Smoking Policies From 2006 to 2012

**DOI:** 10.5888/pcd12.140520

**Published:** 2015-05-14

**Authors:** Rachna D. Chandora, Carrie F. Whitney, Scott R. Weaver, Michael P. Eriksen

**Affiliations:** Author Affiliations: Carrie F. Whitney, Scott R. Weaver, Michael P. Eriksen, Georgia State University, School of Public Health, Atlanta, Georgia.

## Abstract

**Introduction:**

The purpose of this study is to examine the change in smoking policy status among Georgia restaurants and bars from 2006 to 2012 and to identify restaurant and bar characteristics that are associated with allowing smoking.

**Methods:**

Data were obtained from similar cross-sectional indoor air surveys conducted in 2006 and 2012 in Georgia. Both surveys were designed to gather information about restaurant and bar smoking policies. Weighted χ^2^ analyses were performed to identify changes in smoking policy status and other variables from 2006 to 2012. Weighted logistic regression analysis was used to test for significant associations between an establishment’s smoking policy and other characteristics.

**Results:**

The percentage of restaurants and bars in Georgia that allowed smoking nearly doubled, from 9.1% in 2006 to 17.6% in 2012. The analyses also showed a significant increase in the percentage of establishments that allow smoking when minors are present. Having a liquor license was a significant predictor of allowing smoking.

**Conclusion:**

The Smokefree Air Act was enacted in 2005 to protect the health and welfare of Georgia citizens, but study results suggest that policy makers should reevaluate the law and consider strengthening it to make restaurants and bars 100% smokefree without exemptions.

## Introduction

In the United States, smoking and exposure to tobacco smoke kill at least 480,000 people per year (1). Exposure to secondhand smoke in the United States causes approximately 41,000 deaths annually (1) and costs more than $289 billion annually in productivity losses, excess medical care, illness, and death (2). In the state of Georgia, 21.2% of adults smoke, and more than 10,500 adults die each year as a result of tobacco use (3,4). The economic burden of tobacco use in the state is over $3.18 billion in direct health care costs and $3.99 billion in productivity losses annually (5). Additionally, 44.7% of adults in Georgia are exposed to secondhand smoke, ranking Georgia 16th among all states in exposure to secondhand smoke (4).

The most effective way to protect people from the dangers of secondhand smoke is to implement and enforce legislation that requires all indoor public places to be 100% smokefree (6). During the last 3 decades, the United States has made great progress in implementing smokefree policies. In the United States, 77.4% of the population is covered by 100% smokefree restaurant laws and 65.2% of the population is covered by 100% smokefree bar laws; however, Georgia falls far behind the nation, having only 6.2% of the population covered by 100% smokefree restaurant laws and 3.7% of the population covered by 100% smokefree bar laws (7).

The Georgia Smokefree Air Act was signed into law in May 2005. The act prohibits smoking inside most public places and sets guidelines for allowing smoking in and around public establishments (8). The purpose of the act is to limit secondhand smoke exposure among children, adults, and employees and improve the health and comfort of the people of Georgia (9). The law cannot be defined as a 100% smokefree law because it contains provisions that permit establishments to allow smoking if any person under the age of 18 is prohibited from entry to or employment in the establishment and if smoking is allowed only in outdoor areas such as patios or in enclosed private rooms with independent air-handling systems (8).

The primary aims of this study were to examine the change in smoking policy status among bars and restaurants from 2006 through 2012 and to identify characteristics of Georgia restaurants and bars that are associated with allowing smoking.

## Methods

### Overview

Researchers at Georgia State University’s (GSU’s) School of Public Health commissioned the Georgia Smokefree Indoor Air Survey. The cross-sectional surveys, conducted by trained interviewers in 2006 and adapted and repeated in 2012, were administered to a probability sample of restaurant and bar owners or general managers in the state of Georgia. The 2006 and 2012 surveys included more than 50 questions and were designed to gather information about restaurant and bar smoking policies and about owner and manager compliance with and perceptions of the Georgia Smokefree Air Act of 2005. We compared the descriptive characteristics of smoking-allowed establishments in 2006 and 2012 and identified significant changes over time. The surveys were reviewed and approved by the institutional review board of GSU.

### Sampling

We sampled Georgia restaurants and bars identified by the Standard Industrial Classification (SIC) code for type of business and the Federal Information Processing Standard code for state and county location. SIC codes included in the sampling frame were eating places (ie, restaurants, defined as establishments primarily engaged in the retail sale of prepared food and drinks for on-premise or immediate consumption), drinking places (ie, bars, defined as establishments primarily engaged in the retail sale of alcoholic drinks), and restaurants and bars operated by hotels and motels (ie, lodging establishments that serve meals). Survey Sampling International supplied the telephone numbers for the businesses in the sampling frame. A disproportionate stratified random-sampling design was used. The sampling frames were stratified by the following analysis domains: region of the state, whether the establishment was a stand-alone bar, whether the establishment was part of a chain, and whether the establishment was located in a political subdivision with a comprehensive clean indoor air law. The sampling fraction (the ratio of sample size to population size) varied among the strata to achieve an adequate sample size in important domains. The probability sample was unique during each round of survey administration.

### Survey administration

The 2006 survey was administered from May 10, 2006, to June 16, 2006, and the 2012 survey was administered from June 4, 2012, to July 6, 2012. Trained interviewers at the University of Georgia Survey Research Center conducted both surveys via computer-assisted telephone interviews. To ensure high-quality data, approximately one-fourth of all interviews were monitored by supervisors. Additionally, supervisors were present at all times during interviewing. In 2006, 1,150 surveys were completed, exceeding the a priori determined target of 1,000 surveys, and the response rate was 54.2%. In 2012, 843 surveys were completed, exceeding the a priori determined target of 800 surveys, and the response rate was 55.3%.

The 2006 survey was implemented almost 1 year after the Smokefree Air Act was enacted. The initial survey, developed by UGA Survey Research Center staff, in consultation with GSU’s School of Public Health researchers, was designed to gather information about owner and manager experiences with the Georgia Smokefree Air Act of 2005 (10). Modifications were made to the 2012 survey, including the addition of questions about smoking policy status and owner and manager experiences with the law, and questions relevant only to the 2005 implementation of the law were removed.

### Model

The dependent variable examined was smoking policy status of the establishment. Restaurants and bars were categorized either as smokefree or as smoking-allowed facilities on the basis of yes or no answers to the question “Do you allow smoking in your establishment?” Smoking-allowed establishments were further examined by where they allowed smoking: respondents were questioned about their smoking policy in the dining room (dining area in a restaurant), waiting area, bar area (stand-alone bar or bar within a restaurant), and outside area (outdoor dining or drinking area or patio). The following hypothesized predictor variables were examined: cost of a typical meal, smoking policy sign posted at the entrance, having seats for dining outdoors, having a liquor license, having seats for drinking outdoors, employees informed of policy, owner or manager awareness of law, establishment policy change since law implemented, owner or manager opposition to law, and owner or manager belief of exemption from law.

### Analysis

Weighted statistical analyses were conducted by using survey procedures in SAS version 9.4 (SAS Institute Inc). The data were weighted to account for the unequal probability of selection in the sampling design. Weighted χ^2^ tests were conducted to identify changes in smoking policy status and other variables from 2006 to 2012. Weighted logistic regression analysis was performed to assess the unique effect of each predictor variable on the smoking policy status of a bar or restaurant while controlling for the effects of other variables. In all analyses, statistical significance was determined by a probability value of less than .05.

## Results

Most restaurants and bars in Georgia in 2006 and 2012 did not allow smoking, had informed employees of their policy, had posted signs about their smoking policy at their entrance, were aware of the smokefree law, and were not opposed to the law (Table 1). However, the analysis showed that the percentage of restaurants and bars in Georgia allowing smoking nearly doubled from 9.2% in 2006 to 17.6% in 2012, a significant increase. We found a significant increase in the percentage of establishments permitting smoking in designated dining areas and a significant decrease in the percentage of establishments permitting smoking in bar areas (Table 2).

**Table 1:** Characteristics of Restaurants and Bars in Georgia, 2006 and 2012^a^

Variables	2006	2012	*P* Value^b^
	Weighted n = 1,040	Weighted n = 882	
	Weighted Percentage (95% CI)	Weighted Percentage (95% CI)	
Smoking allowed	9.1 (7.4–10.8)	17.6 (14.5–20.6)	<.001
Cost of typical meal is <$10	67.5 (64.3–70.7)	62.8 (58–66.8)	.08
Smoking policy sign posted at entrance	65.7 (62.5–68.9)	73.7 (70.3–77.2)	.001
Have seats for dining outdoors	37.9 (34.8–41.0)	42.0 (38.0–46.0)	.11
Have a liquor license	36.6 (33.5–39.8)	31.0 (27.2–34.6)	.03
Have seats for drinking outdoors	28.2 (25.4–31.1)	34.4 (30.5–38.2)	.01
Employees informed of policy	98.0 (96.9–99.0)	98.6 (97.9–99.3)	.31
Aware of smokefree law	93.0 (91.4–94.6)	83.5 (76.8–87.2)	<.001
Policy changed since smokefree law implemented	20.1 (17.2–22.9)	13.1 (9.7–16.5)	.004
Oppose smokefree law	15.1 (12.7–17.4)	6.2 (4.1–8.3)	<.001
Think they are exempt from smokefree law	6.3 (4.8–7.8)	5.4 (3.3–7.6)	.52

Abbreviations: CI, confidence interval.

a Each variable may not equal total n because of missing data.

b *P* values were determined by using the Rao–Scott χ^2^ test.

**Table 2:** Characteristics of Restaurants and Bars Allowing Smoking in Georgia, 2006 and 2012^a^

Variables	2006	2012	*P* Value^b^
	Weighted n = 95	Weighted n = 154	
	Weighted Percentage (95% CI)	Weighted Percentage (95% CI)	
**Smoking allowed in dining areas**			
Permitted without restriction	28.4 (19.2–37.5)	7.7 (3.7–11.6)	<.001
Permitted in designated areas	20.6 (12.6–28.6)	46.2 (36.3–56.2)	
Not allowed at all	51.1 (40.9–61.2)	46.1 (36.2–56.1)	
**Smoking allowed in waiting areas**			
Permitted without restriction	23.2 (14.7–31.8)	7.4 (3.3–11.5)	<.001
Permitted in designated areas	6.2 (1.7–10.7)	10.2 (5.6–14.9)	
Not allowed at all	49.4 (39.8–59.0)	74.3 (67.7–81.0)	
No waiting area	21.2 (4.3–29.8)	8.0 (3.7–12.3)	
**Smoking allowed in bar areas**			
Permitted without restriction	53.7 (44.6–62.8)	10.2 (5.5–15.0)	<.001
Permitted in designated areas	16.3 (9.3–23.3)	5.7 (2.4–9.0)	
Not allowed at all	24.0 (16.0–32.0)	49.6 (39.9–59.4)	
No bar area	6.0 (1.7–10.4)	34.4 (25.2–43.6)	
**Smoking allowed in outside areas^c^**			
Permitted without restriction	NA	36.4 (27.4–45.5)	NA
Permitted in designated areas	NA	40.8 (31.1–50.5)	
Not allowed at all	NA	13.1 (5.8–20.4)	
No outside areas	NA	9.7 (5.1–14.3)	
**Smoking allowed when minors are present**			
Permitted	18.7 (11.7–25.8)	60.7 (50.7–10.7)	<.001
Not allowed	39.5 (30.1–49.0)	28.5 (19.0–37.9)	
Minors are not permitted	41.7 (32.4–51.0)	10.9 (5.8–16.0)	
**Seats for dining outdoors**			
Seats available	66.7 (56.6–76.8)	67.3 (58.1–76.5)	.93
No seats available	33.3 (23.2–43.4)	32.7 (23.5–41.9)	
**Seats for drinking outdoors**			
Seats available	59.1 (49.4–68.7)	62.5 (53.2–71.9)	.62
No seats available	40.9 (31.1–50.6)	37.5 (28.1–46.8)	
**Have a liquor license**			
Yes	80.2 (72.3–88.0)	52.5 (42.8–62.1)	<.001
No	19.8 (11.9–27.8)	47.5 (37.9–57.2)	

Abbreviation: CI, confidence interval; NA, not available.

a Each variable may not equal total n because of missing data.

b *P* values were determined by using the Rao–Scott χ^2^ test.

c 2006 percentages and *P* value are not available because the question “Is smoking allowed in outside areas” was not included in the 2006 survey.

From 2006 to 2012, we found a reduction in dining areas where smoking was permitted without restriction (from 28.4% in 2006 to 7.7% in 2012) and an increase in dining areas with designated smoking areas (from 20.6% in 2006 to 46.2% in 2012). The analysis indicated a decrease in bar areas where smoking was permitted without restriction (from 53.7% in 2006 to 10.2% in 2012) and bar areas with designated smoking areas (from 16.3% in 2006 to 5.7% in 2012). We also found that more than 75% of smoking-allowed establishments permitted smoking in outside areas, and 22.1% of smoking-allowed restaurants and bars allowed smoking only in outside areas. The percentage change since 2006 could not be assessed because establishments were not asked about their outdoor smoking policy in 2006. Most restaurants and bars that allowed smoking had seats for dining and drinking outdoors. From 2006 to 2012, the percentage of restaurants and bars allowing smoking when minors are present increased significantly (from 18.7% in 2006 to 60.7% in 2012). Additionally, from 2006 to 2012, the proportion of owners and managers that were aware of the smokefree law declined from 93.0% to 83.5%, a significant decrease.

The weighted logistic regression model contained 10 independent variables and was significant (χ^2^_10_ = 63.3, *P* < .001, n = 843), indicating that the model was able to predict establishments that allowed and did not allow smoking. One independent variable, having a liquor license, was significant to the model (Table 3). Establishments that had a liquor license were more than twice as likely to allow smoking as other establishments that did not, controlling for all other factors in the model.

**Table 3:** Weighted Logistic Regression Predicting Likelihood of Allowing Smoking in Restaurants and Bars in Georgia, 2012

Variable	Weighted AOR (95% CI)	*P* Value^a^
**Cost of a typical meal is <$10**		
Yes	1.1 (0.7–1.8)	.74
No	1 [Reference]	
**Smoking policy sign posted at entrance**		
Yes	1.7 (1.0–3.0)	.07
No	1 [Reference]	
**Have seats for dining outdoors**		
Yes	1.5 (0.6–3.5)	.36
No	1 [Reference]	
**Have a liquor license**		
Yes	2.2 (1.2–4.0)	.007
No	1 [Reference]	
**Have seats for drinking outdoors**		
Yes	2.5 (1.0–6.3)	.05
No	1 [Reference]	
**Employees informed of policy**		
Yes	1.1 (.01–10.7)	.94
No	1 [Reference]	
**Aware of smokefree law**		
Yes	1.2 (0.6–2.5)	.68
No	1 [Reference]	
**Policy changed since law implemented**		
Yes	1.0 (0.4–2.2)	.99
No	1 [Reference]	
**Oppose law**		
Yes	1.1 (0.4–3.1)	.88
No	1 [Reference]	
**Think exempt from law**		
Yes	0.9 (0.3–2.8)	.92
No	1 [Reference]	

Abbreviations: AOR, adjusted odds ratio; CI, confidence interval.

a *P* values were determined by using the likelihood ratio Wald χ^2^ test.

## Discussion

The study findings indicate that from 2006 to 2012 the percentage of restaurants and bars in Georgia that allowed smoking nearly doubled. Despite the existence of a smokefree law in Georgia, the proportion of establishments allowing smoking can increase because the law is not comprehensive. It allows restaurants and bars to permit smoking if people under the age of 18 are prohibited and if designated smoking areas are outdoors or in enclosed private rooms with an independent air-handling system. The study results show that restaurant and bar owners have taken advantage of such exemptions in the law. The increase in smoking-allowed establishments may be attributed to the increase in the percentage of establishments permitting smoking in designated dining areas and the large percentage of establishments that permit smoking in outdoor areas.

The study showed a shift from 2006 to 2012 in smoking-allowed restaurants and bars from permitting smoking without restriction to allowing smoking only in designated smoking areas. The percentage of establishments that permit smoking without restriction in dining areas decreased by more than half (from 28.4% in 2006 to 7.7% in 2012), while the percentage of establishments that permit smoking in designated areas (separate area of the establishment where smoking is permitted) more than doubled (from 20.6% in 2006 to 46.2% in 2012). The findings suggest that from 2006 to 2012, restaurants added designated smoking dining areas to accommodate smoking patrons.

The analysis revealed a large decrease in the percentage of establishments that allowed smoking in bar areas. Overall, establishments that allowed smoking in bar areas decreased, from 70.0% in 2006 to 15.9% in 2012. The analysis showed a reduction in unrestricted smoking and designated smoking areas in bar areas and a doubling of establishments not allowing smoking at all in bar areas.

In 2012, 77.2% of smoking-allowed establishments reported allowing smoking in outdoor areas (36.4% permitted smoking without restriction in outdoor areas and 40.8% permitted smoking in designated outdoor areas). Outdoor areas are exempt from the smokefree law; thus, the large percentage of establishments permitting smoking in outside areas, combined with the increase in establishments with outdoor dining and drinking areas, probably contributed to the increase in restaurants and bars that allow smoking. We cannot assess whether the number of establishments allowing smoking in outdoor areas increased because allowing smoking in outdoor areas was a new variable evaluated only in 2012. Further research focusing on the characteristics of restaurants and bars that allow smoking is necessary to fully understand why the number of restaurants and bars allowing smoking increased.

The percentage of establishments that allowed smoking when minors were present increased significantly (from 18.7% in 2006 to 60.7% in 2012). The increase could be attributed to the rise in establishments with designated smoking areas or to the large number of establishments allowing smoking on patios, where minors are allowed without restriction. Among the smoking-allowed establishments that also permitted minors, 12% allowed smoking without restriction and 88% permitted smoking in designated areas. Future studies should examine whether minors are permitted in the designated smoking areas or only in the nonsmoking areas.

The purpose of the Smokefree Air Act is to “preserve and improve the health, comfort, and environment of the people of this State, including children, adults, and employees, by limiting exposure to tobacco smoke” (9). Our study shows that, despite the act, restaurant and bar patrons and employees continue to be exposed to secondhand smoke. Thus, the Smokefree Air Act has not met its primary purpose, and modifications should be made to strengthen the law. Studies have found that partial smokefree laws (or smokefree laws with exemptions), such as Georgia’s law, are not as effective at reducing secondhand smoke exposure, improving air quality, and reducing negative health effects as are 100% smokefree laws (11–15). In addition to reducing secondhand smoke exposure, smokefree policies change social norms about smoking, increase smoking cessation attempts, increase the number of smokers who quit smoking, and reduce cigarette consumption (16).

Policy makers should reassess the Smokefree Air Act and consider strengthening the law to make restaurants and bars 100% smokefree without exemptions. Currently, 35 states have implemented comprehensive smokefree laws in restaurants, and 30 states have implemented comprehensive smokefree laws in restaurants and bars (17). Only 5 states have laws prohibiting smoking in outdoor dining areas, and 4 of those states also prohibit smoking in bar patios (18). When the Smokefree Air Act was enacted in 2005, Georgia was a leader in tobacco control legislation because Georgia was the first major tobacco-producing state to implement smokefree legislation. Now, 9 years later, Georgia has fallen behind most states in regard to smokefree laws. Georgia is 1 of only 15 states that does not have a 100% smokefree restaurant or bar law (17). The gap between Georgia and other states in terms of protection from secondhand smoke will continue to widen if policy makers do not support and implement stronger smokefree laws.

Knowing the characteristics of establishments that currently allow smoking will help policy makers and public health professionals in Georgia and other states without comprehensive smokefree laws craft targeted interventions and outreach. Our study found that establishments that have a liquor license and establishments that have seats for drinking outdoors were more likely to allow smoking. Outreach and educational campaigns should be evidence-based and focus on the body of evidence showing that smokefree laws do not negatively affect restaurants and bars (19–24). These campaigns would alleviate owner and manager concerns about potential adverse economic impact of smokefree policies and raise awareness of the benefits of law. Outreach should be targeted to restaurants and bars that have characteristics associated with allowing smoking.

This study has some limitations. The data were self-reported by restaurant and bar owners and managers. As a result, proprietors may have overreported compliance with smokefree laws, thus introducing response bias, which could affect the validity of the study. Additionally, the question that assesses if the establishment allows smoking asks, “Is smoking allowed anywhere in your establishment?” Owners and managers that allow smoking only in outside areas may misclassify their establishment as a nonsmoking establishment by answering no to this question. Another limitation is that the study design did not allow for testing differences between restaurants and bars. Finally, changes were made to the survey questionnaire in 2012; therefore, researchers were unable to examine changes in certain variables associated with questions that were added to the 2012 questionnaire but were not included in the 2006 questionnaire.

The percentage of restaurants and bars in Georgia allowing smoking nearly doubled from 2006 to 2012. In Georgia, and similar states, policy makers should support comprehensive laws because a large body of research demonstrates that 100% smokefree policies reduce secondhand smoke exposure and improve the health of the public. The findings of this study can help guide the development and implementation of comprehensive smokefree policies for restaurants and bars in Georgia and for other states and localities. Lawmakers and community leaders must act quickly to implement comprehensive smokefree legislation because 100% smokefree laws have the potential to save thousands of lives and millions of dollars in health care expenses annually (25).
